# Giant dog breeds under primary veterinary care in the UK: demography, common disorders and mortality

**DOI:** 10.1186/s40575-026-00152-7

**Published:** 2026-06-26

**Authors:** Dan G. O’Neill, Thomas J. Curtis, Georgia O. J. York, Dave C. Brodbelt, David B. Church, Karolina S. Engdahl

**Affiliations:** 1 Pathobiology and Population Sciences, The Royal Veterinary College, Hawkshead Lane, North Mymms, Hatfield, Herts AL9 7TA UK; 2 Department of Clinical Sciences, Swedish University of Agricultural Sciences, Uppsala, 750 07 Sweden; 3 Clinical Science and Services, The Royal Veterinary College, Hawkshead Lane, North Mymms, Hatfield, Herts AL9 7TA UK

**Keywords:** VetCompass, Electronic health record, EHR, Breed, Primary-care, Purebred, Molossoid, Giant, Dog

## Abstract

**Background:**

The domestic dog is the most phenotypically variable mammalian species on earth but there is limited evidence on the overall health patterns within the subset of giant dog breeds. A VetCompass cohort study design was used to report the demography, one-year prevalence of common disorders and mortality of giant breed dogs under UK primary veterinary care during 2019.

**Results:**

Giant breed dogs from 29 breeds combined to comprise 28,345 (1.26%) from 2,250,417 study dogs. The most common giant breeds were Dogue de Bordeaux, Alaskan Malamute and Akita. Median adult bodyweight overall of giant breed dogs overall was 48.8 kg (IQR 40.4–58.0, range 25.0–100.0). Overall, 73.8% of giant breed dogs had at least one disorder recorded during 2019. The most common precise-level disorders were otitis externa (8.16%, 95% CI: 7.34–8.97), overweight/obesity (8.02%, 95% CI: 7.21–8.83) and aggression (5.56%, 95% CI: 4.88–6.24). The most common grouped-level disorders were dermatological (15.9%, 95% CI: 14.8–17.0), musculoskeletal (13.5%, 95% CI: 12.5–14.5) and aural disorders (12.0%, 95% CI: 11.0–12.9). Among 1,068 giant breed dogs recorded as having died, the median age at death was 8.94 years (IQR 6.96–10.9, range 0.00–16.1). The most common grouped-level precision causes of death were neoplasia (12.4%, 95% CI: 10.4–14.3), collapse (9.46%, 95% CI: 7.70–11.2) and heart disorder (4.12%, 95% CI: 2.93–5.31).

**Conclusions:**

The current results provide some evidence of higher disorder burden and shorter lifespans in giant breed dogs overall compared to the wider population of dogs that raises concerns about negative impacts on both the quality and quantity of life resulting from our human selection for giantism in dogs. These findings suggest value from considering setting international welfare-based limits for height and bodyweight exaggerations selected in giant dogs.

## Lay summary

The domestic dog is the single mammalian species with the most variation in body shape. The UK Royal Kennel Club (RKC) recognises 227 distinct breeds while data from VetCompass on dogs under UK veterinary care includes 800 distinct breeds in the wider population of dogs. Humanity has artificially selected several of these breeds for large body size ie giantism, originally to enhance functionality as watch, shepherd, draught and fighting dogs and more latterly as show and companion dogs. However, there remains limited evidence on the health and welfare issues related to giantism in dogs. The literature suggests some predispositions in giant breeds towards musculoskeletal and cancer disorders and also to shorter lifespans compared to non-giant breed dogs. To fill these gaps, the current study aimed to report the demography of giant breed dogs in the UK and to identify their most common disorders and their life expectancy. The study included all dogs under UK primary veterinary care at clinics participating in the VetCompass Programme during 2019. All dogs were characterised by breed. A list of giant breeds was derived with assistance from the RKC. The clinical records of a random sample from all available giant breed dogs were manually reviewed and information was extracted on all disorders during 2019 and on all deaths recorded at any date in the clinical records. Demographic information was also extracted on the giant breed dogs, including date of birth and bodyweight. The proportion of giant breed dogs diagnosed with each disorder during 2019 and the age at death for all dogs that died were reported. From 2,250,417 dogs overall in the study, there were 28,345 (1.26%) giant breed dogs spread across 29 different breeds. Among these 29 breeds, the most common giant breeds were Dogue de Bordeaux (n = 5,156, 18.20%), Alaskan Malamute (n = 3,702, 13.10%) and Akita (n = 2,901, 10.20%). The median adult bodyweight by breed varied from Hungarian Kuvasz (31.4kg) as the lowest to Saint Bernard (65.1kg) as the heaviest. From assessing clinical records of a random sample of 4,316/28,345 (15.2%) giant breed dogs, the most common specific disorders recorded were otitis externa (prevalence 8.16%), overweight/obesity (prevalence 8.02%) and aggression (prevalence 5.56%). The median age of dogs with the 31 most common precise-level diagnoses varied from 3.03 years for diarrhoea to 10.9 years for collapsed. After combining the disorders into groups, the most common disorder groups were skin (prevalence 15.9%), musculoskeletal (prevalence 13.5%) and ear disorders (prevalence 12.0%). From 1,068 (24.75%) of the giant breed dogs recorded as dying during the available clinical records, the average age at death was 8.94 years. Females (average longevity 9.31 years) lived statistically longer than males (average longevity 8.49 years). The average age at death ranged across the giant breeds from 4.77 years in the Tibetan Mastiff to 12.7 years in the Black Russian Terrier. Of 993 (93.0%) deaths with a recorded method of death, 872 (87.8%) deaths involved euthanasia, and 121 (12.2%) deaths were unassisted. The current results provide some evidence of higher disorder burden and shorter lifespans in giant breed dogs overall compared to the wider population of dogs that raises concerns about negative impacts on both the quality and quantity of life resulting from our human selection for giantism in dogs. These findings suggest value from considering setting international welfare-based limits for height and bodyweight exaggerations selected in giant dogs.

## Background

The domestic dog is the most phenotypically variable mammalian species on earth, with the UK Royal Kennel Club (RKC) recognising 225 distinct breeds among the pedigree subset of dogs, while 800 dog distinct breeds are recognised in the wider general population of dogs in the UK [1–3]. The domestic dog (*Canis lupus familiaris*) is considered the first mammalian species to become domesticated, around 30,000–15,000 years ago [4, 5]. Over the past few thousands of years, increasing body size was artificially selected by humans to enhance functionality in dogs such as watch, shepherd, draught and fighting while still remaining within the limits of good health and longevity to support useful working function [6–9]. However, with the invention of ‘breed’ as a concept and the popularity of dog showing over the past century, the requirement for good health and longevity as key breeding criteria was relaxed, enabling prioritisation of aesthetics and exaggeration to promote selection pressures towards even greater body size and giantism [10, 11]. This human drive towards evermore exaggerated features in dogs is well described by the American Kennel Club as ‘Some people live by the motto “bigger is better,” and it extends to their dogs, too’ [12]. There is now growing awareness of the existence of a health and welfare line between benign and malign exaggeration in dogs, with the latter representing extreme conformation where bigger may not be better [10, 11, 13]. However, despite the growing evidence on the harms of extreme conformation in dogs, there remains limited benchmarking data on baseline health and longevity of giant dogs that can assist wider evidence-based discussions on limits that could be applied to giantism exaggeration [14].

Despite the strict breed standards applied to define individual pedigree dog breeds registered by kennel clubs worldwide, paradoxically there are no universally agreed height or bodyweight criteria for being a giant dog breed. However, there seems to be tacit agreement that breeds with a typical adult bodyweight greater than 45 kg (99 lbs) are considered as giant [12, 15, 16]. Additionally, despite humanity’s recent fascination with breed as an emotive canine concept, there still appear to be no defined lists of giant dog breeds along with published information on their relative and absolute popularity and adult bodyweights among the wider population of dogs owned in the UK [17]. Such information is critical to understand the presence or scale of any welfare issues that may relate to giantism among domestic dogs as a species.

The literature provides some evidence of predisposition in giant breed dogs to some musculoskeletal, cardiorespiratory and neoplastic disorders, with higher growth rates implicated in increased oxidative damage during early life as a proposed predisposing factor [18]. Musculoskeletal disorder predisposition in giant breeds is reported for hip dysplasia, elbow dysplasia and osteoarthritis in general, although other environmental factors associated with being giant such as diet or exercise may confound these associations [19–23]. Cardiorespiratory disorder predisposition in giant breeds is suggested for laryngeal paralysis [24, 25] and dilated cardiomyopathy [26, 27]. Whether giant breed dogs are predisposed to neoplasia overall remains uncertain but there is strong evidence of giant breed predisposition to osteosarcoma [28–30]. In addition, there are reported predispositions in some individual giant breeds to specific neoplasia types, such as histiocytosis in Bernese Mountain Dogs [31] and squamous cell carcinoma of the digit in large black Giant Schnauzers [32, 33]. Undesirable behaviours such as aggression or separation related behaviour are increasingly recognised as key welfare concerns for both dogs and their in-contact humans [34]. Although the evidence is mixed, the current literature does not show higher absolute probability of aggression in giant breeds compared to smaller-sized dogs, but the implications from aggression for the dogs and their in-contact humans do seem higher for giant breed dogs [35, 36]. However, much of the current evidence on disorder risk in giant breed dogs was based on studies of individual disorders or case series that make it challenging to interpret either the prevalence or predisposition for giant dog breeds overall.

Life expectancy is widely accepted in human and companion animal demography as a useful proxy indicator of the general health of the population [37, 38]. Low life expectancy implies that events leading to mortality occur earlier, on average, and therefore such information can raise awareness of opportunities for positive welfare change [38]. There is a strong evidence base supporting diminishing life expectancy as body size increases in dogs [38–41]. These shorter lifespans may be linked to the move from natural selection to artificial selection following the domestication of dogs, with physical giantism exaggeration requiring rapid and excessive growth that may bring more rapid ageing as a consequence [42] and promote earlier development of multiple degenerative disorders [43–45]. A better understanding of the life expectancy and the causes of mortality of dogs from the wider population of giant breeds could contribute to identifying opportunities to limit any negative welfare effects of giantism and even reframe the welfare-acceptable boundaries of exaggeration currently in common use.

With this background, the current study aimed to report the demography of giant breed dogs in the UK and to identify their most common causes of morbidity and mortality as well as reporting their life expectancy. This information could contribute to a greater evidence base to support ongoing discussion and reforms aimed at limiting any negative health and welfare effects from extreme conformation in dogs [11].

## Materials and methods

The study population included all dogs under UK primary veterinary care at clinics participating in the VetCompass Programme during 2019. Dogs under veterinary care were defined as having at least one electronic health record [EHR] (free-text clinical note, treatment, or bodyweight) recorded during 2019. VetCompass data fields available for the current study included fixed variables of unique animal identifier, species, breed, date of birth, sex and neuter status along with time-varying variables of bodyweight, free-form text clinical notes and treatment with relevant dates [46–48].

A list of giant breeds was derived with assistance from the RKC (Table 1). The bodyweight, sex, neuter status and age for dogs of each of these giant breeds under veterinary care during 2019 were described. *Adult Bodyweight* (kg) described the mean bodyweight recorded from all bodyweight data for dogs aged over 18 months and was categorised into 4 groups (< 40.0 kg, 40 to < 50 kg, 50 to < 60 kg, ≥ 60 kg). All-age bodyweight (kg) described the mean bodyweight of all available bodyweights for each dog. *Neuter* described the status of the dog (entire or neutered) at the final EHR. *Age* (years) described the age at the final date under veterinary care during 2019 (December 31^st^, 2019) and was categorised into 6 groups (≤ 2.0, 2.0 to < 4.0, 4.0 to < 6.0, 6.0 to < 8.0, 8.0 to < 10.0 and ≥ 10.0). Breeds were categorised as Molossoid/non-Molossoid based on the classification by the Fédération Cynologique Internationale [49], with the Anatolian Karabash Shepherd Dog additionally classified as Molossoid.

A cohort study design was used to estimate the one-year period prevalence of the most frequently diagnosed disorders of giant breed dogs from a population of 2,250,417 dogs across all breeds under primary veterinary care during 2019 at VetCompass participating practices. For the current study, a disorder was defined as any deviation from full health recorded in the EHR, with this definition aligned with the National Institutes of Health (NIH) definition of a disorder as ‘an abnormal condition that affects the body’s function but may or may not have specific signs and symptoms’, and with there being ‘many different types of disorders, including physical, mental, emotional, behavioral, genetic, and functional disorders’ [50]. For reporting disorder prevalence, sample size calculations estimated that 3,012 dogs were needed to report on a disorder with 2.0% expected prevalence with 95% confidence level and 0.50% margin of error [51]. Ethical approval was given by the RVC Social Science Research Ethical Review Board (SSRERB) (reference number SR2018-1652).

The EHRs of a random sample of 4,316 dogs from all available giant breed dogs were manually reviewed in detail to extract the most definitive diagnoses recorded for all disorders recorded as existing during 2019 and to link these to the most appropriate VeNom term as previously described [47]. The extracted diagnosis terms were mapped to a dual hierarchy of precision for analysis: precise-level precision and grouped-level precision [47]. Precise-level terms described the original extracted terms at the maximal diagnostic precision recorded within the clinical notes (e.g. *inflammatory bowel disease* remained as *inflammatory bowel disease*). Grouped-level terms mapped the original diagnosis terms to a general level of diagnostic precision (e.g. *inflammatory bowel disease* mapped to *enteropathy)*. Disorders described within the clinical notes using presenting sign terms (e.g. ‘vomiting’ or ‘vomiting and diarrhoea’) without a formal clinical diagnostic term were included using the first sign listed (e.g. vomiting). Elective (e.g. neutering) or prophylactic (e.g. vaccination) clinical events were excluded. No distinction was made between pre-existing and incident disorder presentations. Mortality data (recorded cause, date and method of death) were extracted on all deaths at any date during the available EHRs.

Following data checking for internal validity and cleaning in Excel (Microsoft Office Excel 2013, Microsoft Corp.), analyses were conducted using R version 4.2.1 [52].

Annual proportional birth rates described the relative proportion of giant breed dogs compared with all dogs that were born in each year from 2013 to 2019 from the overall cohort of dogs under veterinary care in 2019 and were illustrated in a figure generated with the R package ggplot2 [53]. Bodyweight data at any age were used to generate bodyweight growth curves for males and females up to the age of 15 years for the six most numerous giant breeds. These were plotted with a cross median line (up to the age of 10 years) with the R package ggplot2 and combined with ggpubr [54, 55].

One-year (2019) period prevalence values with 95% confidence intervals (CI) were reported to describe the probability of diagnosis of the most common disorders recorded as occurring at least once during 2019. The CI estimates were derived from standard errors based on approximation to the normal distribution (Wald CI, binom_approx) for disorders with ten or more events [56] or the Wilson approximation (binom.wilson) method for disorders with fewer than ten events [57], using the R package epitools [58]. Prevalence values were reported overall, separately for males and females and Molossoid/non-Molossoid breeds. Median age (years) as defined above was reported for the most common precise-level and grouped-level diagnoses. The 10 most common disorders were identified at grouped-level precision in dogs in three age bands (< 2 years, 2–7 years, and > 7 years). The prevalence of these disorders through life up to the age 14 years is presented in a figure generated with the R packages ggplot2, cowplot, and ggpubr [53, 54, 59], using loess curves. The Shapiro-Wilk test and visual assessment of histograms were used to assess normality of continuous variables. The two-proportion z-test was used to compare proportions, the chi-square test to compare categorical variables, and the Mann-Whitney U test to compare continuous variables [56]. No adjustment for multiple comparisons was applied to the p-values [60]. Statistical significance was set at the 5% level.

## Results

### Demography

The study population of 2,250,417 dogs under veterinary care during 2019 in the UK included 28,345 (1.26%) giant breed dogs spread across 29 different breeds. The number of dogs within each giant breed and their median adult bodyweight is presented in Table 1. The most common giant breeds were Dogue de Bordeaux (*n* = 5,156, 18.20%), Alaskan Malamute (*n* = 3,702, 13.10%) and Akita (*n* = 2,901, 10.20%). The median adult bodyweight by breed varied from Hungarian Kuvasz (31.4 kg) as the lowest to Saint Bernard (65.1 kg) as the heaviest.

**Table 1:** Demography of giant dog breeds under primary veterinary care at practices participating in the VetCompass™ Programme in the UK from January 1st to December 31st, 2019

Breed	Molossoid status	Number of dogs	% of all 28,345 giant breed dogs	Percent of all 2,250,417 dogs overall	Median breed bodyweight (kg)	Median bodyweight - Female (kg)	Median bodyweight - Male (kg)
Akita	Non-Molossoid	2,901	10.2	0.13	40.1	37.6	42.7
Alaskan Malamute	Non-Molossoid	3,702	13.1	0.16	40.7	38.0	43.6
American Akita	Non-Molossoid	2,090	7.37	0.09	39.3	36.4	42.0
Anatolian Karabash Shepherd Dog	Molossoid	129	0.46	0.01	54.0	47.5	54.7
Bernese Mountain Dog	Non-Molossoid	1,513	5.34	0.07	45.3	42.5	48.8
Black Russian Terrier	Non-Molossoid	194	0.68	0.01	48.7	45.2	51.1
Bloodhound	Non-Molossoid	139	0.49	0.01	46.7	41.9	50.7
Borzoi	Non-Molossoid	165	0.58	0.01	37.2	33.4	41.9
Bull Mastiff	Molossoid	1,802	6.36	0.08	49.3	45.3	53.3
Dogue de Bordeaux	Molossoid	5,156	18.20	0.23	51.2	47.5	55.1
Estrela Mountain Dog	Molossoid	59	0.21	< 0.01	43.2	41.6	46.0
Giant Schnauzer	Non-Molossoid	509	1.80	0.02	39.2	36.2	42.0
Great Dane	Molossoid	2,623	9.25	0.12	60.5	56.0	64.9
Great Swiss Mountain Dog	Non-Molossoid	93	0.33	< 0.01	51.7	47.1	54.5
Hungarian Kuvasz	Non-Molossoid	15	0.05	< 0.01	31.4	29.8	31.4
Irish Wolfhound	Non-Molossoid	367	1.29	0.02	64.0	61.0	68.0
Komondor	Non-Molossoid	19	0.07	< 0.01	48.4	44.1	54.8
Leonberger	Molossoid	706	2.49	0.03	56.9	52.7	60.6
Mallorquin Mastiff	Molossoid	12	0.04	< 0.01	53.9	-	53.9
Mastiff	Molossoid	1,341	4.73	0.06	48.8	45.5	52.3
Neapolitan Mastiff	Molossoid	308	1.09	0.01	59.8	55.5	65.3
Newfoundland	Molossoid	2,174	7.67	0.10	58.8	54.6	62.6
Otterhound	Non-Molossoid	61	0.22	< 0.01	41.8	37.1	45.2
Pyrenean Mastiff	Molossoid	5	0.02	< 0.01	53.5	51.8	67.2
Pyrenean Mountain Dog	Molossoid	267	0.94	0.01	50.1	47.6	53.0
Saint Bernard	Molossoid	1,466	5.17	0.07	65.1	59.7	70.0
Scottish Deerhound	Non-Molossoid	267	0.94	0.01	40.6	37.5	43.6
Tibetan Mastiff	Molossoid	216	0.76	0.01	47.3	43.6	51.6
Turkish Kangal	Molossoid	46	0.16	0.00	58.9	49.2	60.0

**Fig. 1:**
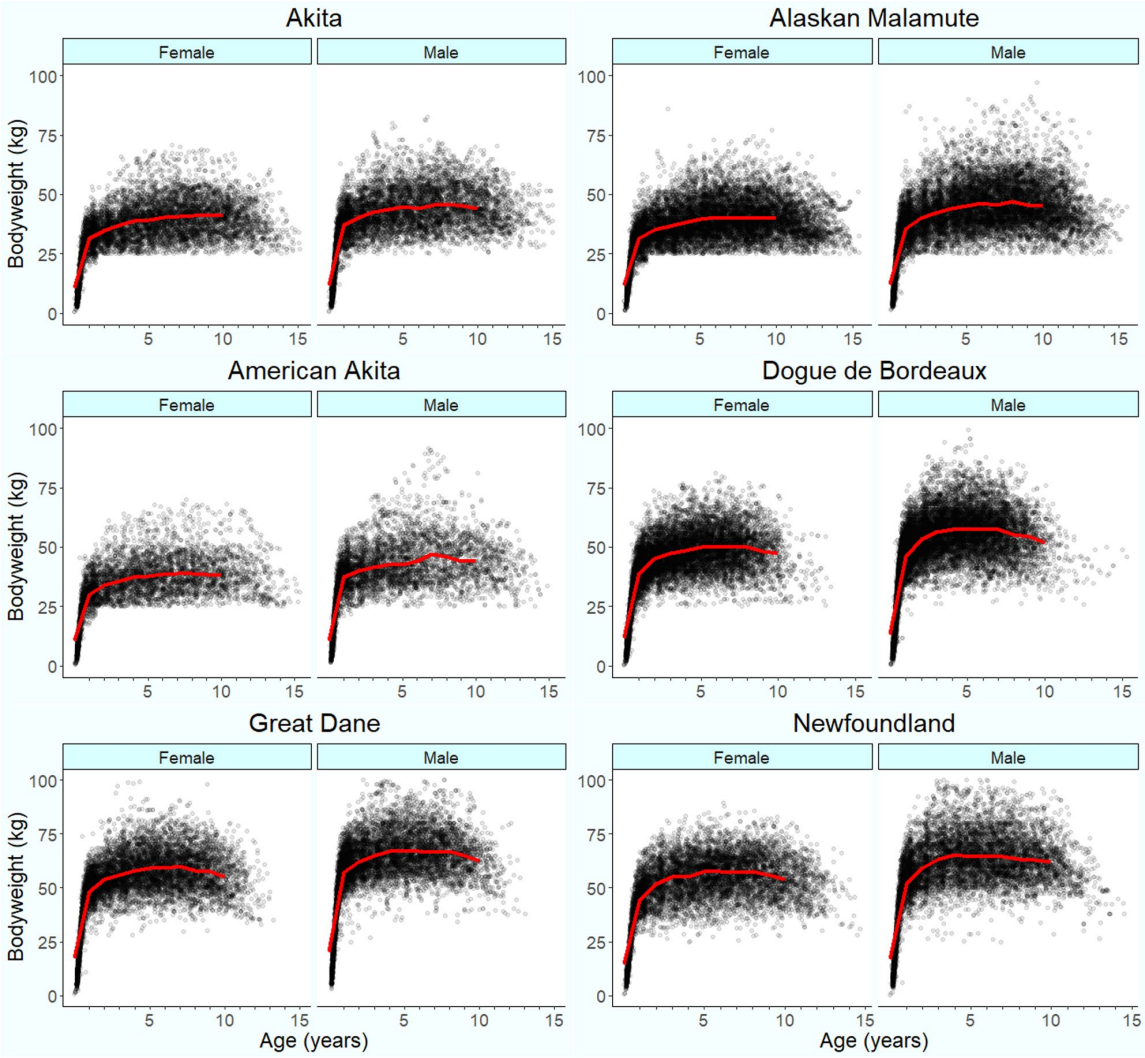
Bodyweight for females and males at different ages (with a cross median line shown in red) of the six most numerous giant dog breeds under UK primary veterinary care from January 1^st^ to December 31^st^, 2019, at practices participating in the VetCompass™ Programme

Of the giant breed dogs with information available, 13,637 (48.6%) were female and 8,613 (30.7%) were neutered (Table 2). Proportional neuter status was statistically significantly higher in females (32.7%) than males (28.9%) (chi-square test: *P* < 0.001). The overall median age was 4.32 years (interquartile range (IQR) 1.84–7.37, range 0.02–23.5). The median adult bodyweight was 48.8 kg (IQR 40.4–58.0, range 25.0–100.0). Adult males (52.4 kg, IQR 43.6–62.0, range 25.0–100.0) were statistically significantly heavier than adult females (45.1 kg, IQR 37.9–53.7, range 25.0–99.0) (Mann-Whitney U test: *P* < 0.001). The adult bodyweight for Molossoid breeds (median 55.0 kg, IQR 47.4–63.0, range 25.0–100.0) was statistically significantly higher than for non-Molossoid breeds (median 41.3 kg, IQR 35.7–48.0, range 25.0–97.2) (Mann-Whitney U test: *P* < 0.001). All-life bodyweight was statistically significantly higher in males (median 44.9 kg, IQR 34.4–55.6, range 0.52–99.8) than females (median 39.4 kg, IQR 30.6–48.6, range 0.40–99.0) (Mann-Whitney U test: *P* < 0.001). Bodyweight growth curves for the top six most numerous giant breeds are presented in Fig. 1. Proportional completeness for the demographic variables were sex 98.9%, neuter 99.2%, adult bodyweight 71.4% and age 99.2%.

**Table 2:** Demography of dogs from 29 giant breeds under primary veterinary care at practices participating in the VetCompass™ Programme in the UK from January 1st to December 31st, 2019.

Variable	Category	Female (%)*	Male (%)*	Total (%)*
Neuter	Neutered	4,456 (32.7)	4,157 (28.9)	8,613 (30.7)
Adult bodyweight (kg)	< 40 kg	3,139 (31.8)	1,630 (15.8)	4,781 (23.6)
	40 to < 50 kg	3,241 (32.9)	2,746 (26.6)	6,004 (29.7)
	50 to < 60 kg	2,260 (22.9)	2,861 (27.7)	5,140 (25.4)
	≥ 60 kg	1,224 (12.4)	3,076 (29.8)	4,320 (21.3)
Age (years)	< 2	3,454 (25.5)	3,957 (27.7)	7,593 (27.0)
	2 to < 4	2,648 (19.6)	2,909 (20.3)	5,583 (19.9)
	4 to < 6	2,337 (17.3)	2,445 (17.1)	4,813 (17.1)
	6 to < 8	2,120 (15.7)	2,162 (15.1)	4,296 (15.3)
	8 to < 10	1,655 (12.2)	1,578 (11.0)	3,251 (11.6)
	≥ 10	1,318 (9.74)	1,245 (8.71)	2,582 (9.18)

*Counts cover dogs with available data

**Fig. 2:**
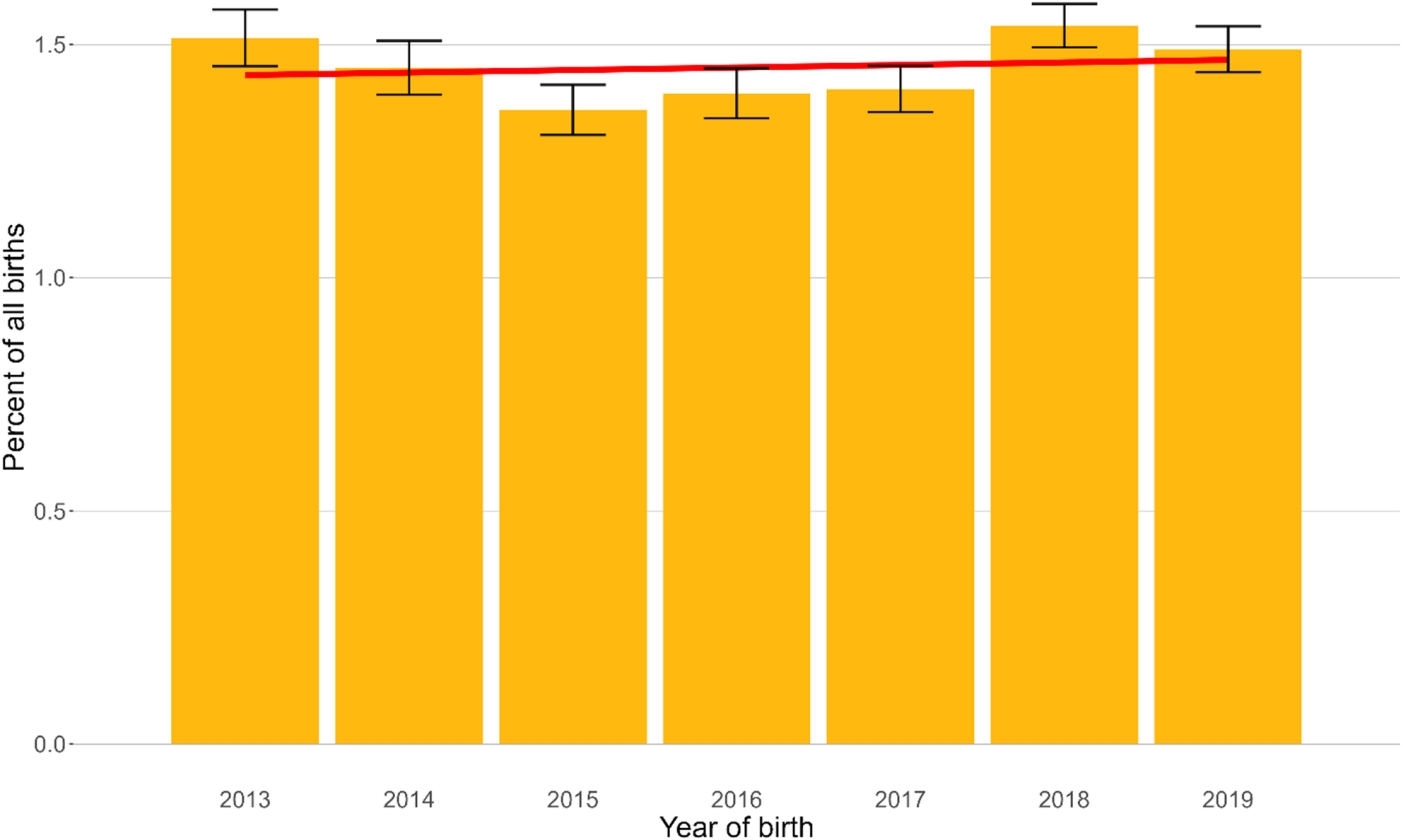
Annual proportional birth rates (2013–2019) (with linear trend line shown in red) and 95% confidence intervals for dogs from giant breeds (*n =* 28,345) among all dogs (*n =* 2,250,417) under UK primary veterinary care from January 1^st^ 2019 to December 31^st^, 2019 at practices participating in the VetCompass™ Programme

Annual proportional birth rates showed a stable breed popularity during 2013–2019, from 1.51% of all dogs born in 2013 to 1.49% in 2019 (Fig. 2).

### Disorder prevalence

The EHRs from a random sample of giant breed dogs (4,316/28,345, 15.2%) were manually reviewed and information on all disorders recorded during 2019 was extracted. Of these sampled giant breed dogs, 3,185/4,316 (73.8%) had at least one disorder recorded during 2019, while the remainder received only prophylactic care or no active veterinary care during 2019. There were 7,423 unique disorder events reported for these 4,316 dogs during 2019, with a median annual disorder count of 1 (IQR 0–2, range 0–13) disorder per dog. The annual disorder count per dog did not differ significantly between females (median count 1, IQR 0–2, range 0–13) and males (median count 1, IQR 0–3, range 0–13) (Mann-Whitney U test, *P* = 0.144).

There were 536 unique precise-level disorder terms reported during 2019. The most common precise-level disorders were otitis externa (*n* = 352, prevalence 8.16%, 95% CI: 7.34–8.97), overweight/obesity (*n* = 346, prevalence 8.02%, 95% CI: 7.21–8.83) and aggression (*n* = 240, prevalence 5.56%, 95% CI: 4.88–6.24) (Table 3). Among the 31 most common precise-level disorders, females had a higher probability of urinary incontinence and cruciate disease, while males had a higher probability of aggression, moist dermatitis, non-wound-related post-operative complications and pruritus (two-proportion z-test: *P* < 0.05). The median age of dogs with the 31 most common precise-level diagnoses varied from 3.03 years for diarrhoea to 10.9 years for collapsed (Table 3).

**Table 3:** Prevalence of the most common disorders at *precise-level diagnostic precision* in giant dog breeds (*n* = 4,316) under primary veterinary care at practices participating in the VetCompass™ Programme in the UK from January 1^st^ to December 31^st^, 2019. *CI confidence interval.

Precise-level disorder	No.	Prevalence % (95% CI*)	Female prevalence %	Male prevalence %	*P*-value**	Median age (years) of affected dogs
Otitis externa	352	8.16 (7.34–8.97)	7.39	8.98	0.067	4.14
Overweight/obesity	346	8.02 (7.21–8.83)	8.17	7.99	0.875	5.94
Aggression	240	5.56 (4.88–6.24)	3.60	7.41	**< 0.001**	4.76
Dental disease	238	5.51 (4.83–6.20)	6.18	4.94	0.089	6.42
Overgrown nail(s)	232	5.38 (4.70–6.05)	5.89	4.80	0.132	4.73
Diarrhoea	207	4.80 (4.16–5.43)	4.28	5.34	0.121	3.03
Periodontal disease	185	4.29 (3.68–4.89)	4.33	4.22	0.921	7.30
Osteoarthritis	173	4.01 (3.42–4.59)	4.04	4.00	> 0.999	9.37
Lameness	141	3.27 (2.74–3.80)	3.31	3.28	> 0.999	4.45
Disorder undiagnosed	137	3.17 (2.65–3.70)	3.02	3.32	0.627	9.41
Vomiting	129	2.99 (2.48–3.50)	2.87	3.14	0.664	3.59
Aural discharge	116	2.69 (2.21–3.17)	2.68	2.74	0.972	3.85
Moist dermatitis	115	2.66 (2.18–3.14)	1.22	4.04	**< 0.001**	4.33
Anal sac impaction	105	2.43 (1.97–2.89)	2.48	2.33	0.833	4.72
Conjunctivitis	105	2.43 (1.97–2.89)	2.29	2.56	0.630	3.23
Behaviour disorder	100	2.32 (1.87–2.77)	2.63	2.07	0.266	3.38
Wound	94	2.18 (1.74–2.61)	2.04	2.33	0.584	3.66
Anxious/distressed	91	2.11 (1.68–2.54)	2.38	1.89	0.307	3.92
Post-operative complication – wound related	89	2.06 (1.64–2.49)	1.99	2.11	0.873	4.08
Collapsed	87	2.02 (1.60–2.44)	1.99	2.07	0.954	10.9
Skin cyst	85	1.97 (1.55–2.38)	2.04	1.93	0.879	6.41
Pyoderma	81	1.88 (1.47–2.28)	1.61	2.16	0.227	3.25
Post-operative complication – non-wound related	68	1.58 (1.20–1.95)	1.07	2.07	**0.013**	3.24
Alopecia	66	1.53 (1.16–1.90)	1.46	1.62	0.769	5.50
Allergic skin disorder	64	1.48 (1.12–1.84)	1.51	1.44	0.948	3.99
Entropion	63	1.46 (1.10–1.82)	1.51	1.44	0.948	3.73
Pruritus	63	1.46 (1.10–1.82)	1.02	1.84	**0.034**	3.97
Cruciate disease	62	1.44 (1.08–1.79)	1.85	1.03	**0.034**	6.01
Thin	60	1.39 (1.04–1.74)	1.12	1.62	0.206	4.12
Urinary incontinence	60	1.39 (1.04–1.74)	2.48	0.40	**< 0.001**	8.42
Dermatitis	57	1.32 (0.98–1.66)	1.17	1.48	0.445	4.10

** two-proportion z-test to compare prevalence between males and females with P-values < 0.05 shown in bold

There were 65 unique grouped-level disorder terms reported during 2019. The most common grouped-level disorders were dermatological (*n* = 686, prevalence 15.9%, 95% CI: 14.8–17.0), musculoskeletal (*n* = 583, prevalence 13.5%, 95% CI: 12.5–14.5) and aural disorders (*n* = 516, prevalence 12.0%, 95% CI: 11.0–12.9) (Table 4). Among the 21 most common grouped-level disorders, females had a higher probability of urinary system disorders and neoplasia, while males had a higher probability of dermatological disorders, behavioural disorders, and enteropathy (*P* < 0.05, two-proportion z-test). The median age of dogs with the most common grouped-level disorders ranged from 3.64 years for enteropathy to 10.9 years for collapsed (Table 4).

**Table 4:** Prevalence of the most common disorders at *grouped-level diagnostic precision* in giant dog breeds (*n* = 4,316) under primary veterinary care at practices participating in the VetCompass™ Programme in the UK from January 1^st^ to December 31^st^, 2019.

Grouped-level disorder	No.	Prevalence % (95% CI*)	Female prevalence %	Male prevalence %	*P*-value**	Median age (years) of affected dogs
Dermatological	686	15.9 (14.8–17.0)	13.6	18.2	**< 0.001**	4.18
Musculoskeletal	583	13.5 (12.5–14.5)	12.7	14.3	0.155	6.84
Aural	516	12.0 (11.0–12.9)	11.1	12.9	0.071	4.15
Enteropathy	511	11.8 (10.9–12.8)	10.8	13.0	**0.028**	3.64
Behavioural	463	10.7 (9.80–11.7)	9.24	12.2	**0.002**	4.11
Dental	409	9.48 (8.60–10.4)	10.4	8.71	0.074	6.58
Overweight/obesity	346	8.02 (7.21–8.83)	8.17	7.99	0.875	5.94
Ophthalmological	297	6.88 (6.13–7.64)	6.81	6.96	0.893	4.02
Mass	288	6.67 (5.93–7.42)	7.20	6.29	0.259	7.11
Claw/nail	287	6.65 (5.91–7.39)	7.00	6.20	0.316	4.76
Neoplasia	270	6.26 (5.53–6.98)	7.10	5.52	**0.039**	8.17
Traumatic injury	178	4.12 (3.53–4.72)	3.94	4.31	0.594	3.79
Complication associated with clinical care	145	3.36 (2.82–3.90)	2.92	3.77	0.143	3.69
Respiratory tract	142	3.29 (2.76–3.82)	3.16	3.41	0.708	5.10
Disorder undiagnosed	137	3.17 (2.65–3.70)	3.02	3.32	0.627	9.41
Heart	130	3.01 (2.50–3.52)	3.06	2.96	0.918	6.75
Anal sac	120	2.78 (2.29–3.27)	3.02	2.51	0.364	4.82
Female reproductive (females only)	92	2.13 (1.70–2.56)	4.47	0.00	**-**	4.62
Urinary	91	2.11 (1.68–2.54)	2.97	1.35	**< 0.001**	6.11
Collapsed	87	2.02 (1.60–2.44)	1.99	2.07	0.954	10.9
Thin	80	1.85 (1.45–2.26)	1.56	2.11	0.218	5.02

*CI confidence interval. ** two-proportion z-test to compare prevalence between males and females with P-values < 0.05 shown in bold

**Fig. 3:**
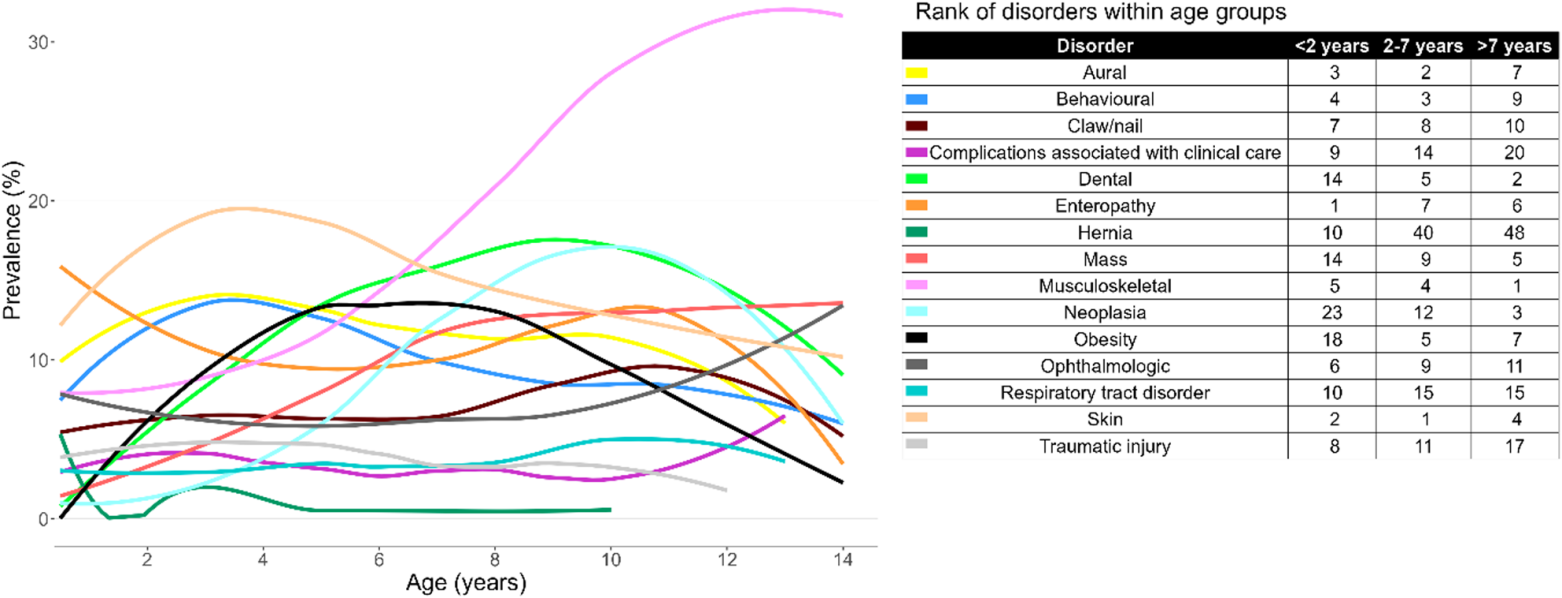
Figure shows the annual prevalence of the 10 most common grouped-level disorders with smoothed lines between the years. The corresponding table shows their rank by prevalence within each of three age bands (under 2 years *n* = 1,151, 2–7 years, n *=* 1,964, over 7 years *n* = 1,177) for giant dog breeds under primary veterinary care at UK practices participating in the VetCompass™ Programme from January 1^st^ to December 31^st^, 2019

The annual prevalence of the top 10 most common grouped-level disorders along with their prevalence rank within three age bands (< 2 years, 2–7 years, and > 7 years) is presented in Fig. 3. There were 1,151 dogs aged under 2 years, 1,964 dogs aged from 2 to 7 years, and 1,177 dogs aged over 7 years. The prevalence of enteropathy, hernia, masses, neoplasia, overweight/obesity, and aural, behavioural, dental, musculoskeletal and skin disorders (10/15 of the disorders, 66.7%) varied significantly between the age groups (chi-square test, *P* < 0.05).

## Molossoid vs. non-molossoid breeds

The random sample of 4,316 dogs included 2,468 (57.2%) dogs of Molossoid breeds and 1,848 (42.8%) dogs of non-Molossoid breeds (Table 1). Among the 31 most common precise-level disorders of giant breed dogs overall, Molossoid breeds had a statistically significantly higher prevalence of 5/31 (16.13%) (alopecia, skin cyst, lameness, otitis externa, aural discharge, entropion) and statistically significantly lower prevalence of 6/31 (19.35%) (overweight/obesity, diarrhoea, aggression, dental disease, periodontal disease) compared with non-Molossoid breed dogs. No statistically significant difference in prevalence between Molossoid breed and non-Molossoid breed dogs was detected in the remaining 20/31 (64.52%) disorders (Table 5).

**Table 5:** Prevalence of the most common disorders at *precise-level diagnostic precision* in giant dog breeds overall broken down by Molossoid (*n* = 2,468) and non-Molossoid (*n* = 1,848) dog breeds under primary veterinary care at practices participating in the VetCompass™ Programme in the UK from January 1st to December 31st, 2019

Precise-level disorder	Molossoid no. affected	Molossoid prevalence % (95% CI*)	Non-Molossoid no. affected	Non-Molossoid prevalence % (95% CI*)	*P*-value**
Otitis externa	265	10.7 (9.52-12.0)	87	4.71 (3.74–5.67)	**< 0.001**
Overweight/obesity	167	6.77 (5.78–7.76)	179	9.69 (8.34-11.0)	**0.001**
Aggression	103	4.17 (3.38–4.96)	137	7.41 (6.22–8.61)	**< 0.001**
Dental disease	96	3.89 (3.13–4.65)	142	7.68 (6.47–8.90)	**< 0.001**
Overgrown nail(s)	124	5.02 (4.16–5.89)	108	5.84 (4.77–6.91)	0.265
Diarrhoea	103	4.17 (3.38–4.96)	104	5.63 (4.58–6.68)	**0.032**
Periodontal disease	79	3.20 (2.51–3.90)	106	5.74 (4.68–6.80)	**< 0.001**
Osteoarthritis	100	4.05 (3.27–4.83)	73	3.95 (3.06–4.84)	0.928
Lameness	93	3.77 (3.02–4.52)	48	2.60 (1.87–3.32)	**0.040**
Disorder undiagnosed	87	3.53 (2.80–4.25)	50	2.71 (1.97–3.45)	0.152
Vomiting	74	3.00 (2.33–3.67)	55	2.98 (2.20–3.75)	> 0.999
Aural discharge	98	3.97 (3.20–4.74)	18	0.97 (0.53–1.42)	**< 0.001**
Moist dermatitis	66	2.67 (2.04–3.31)	49	2.65 (1.92–3.38)	> 0.999
Anal sac impaction	55	2.23 (1.65–2.81)	50	2.71 (1.97–3.45)	0.364
Conjunctivitis	51	2.07 (1.51–2.63)	54	2.92 (2.15–3.69)	0.088
Behaviour disorder	51	2.07 (1.51–2.63)	49	2.65 (1.97–3.38)	0.245
Wound	56	2.27 (1.68–2.86)	38	2.06 (1.41–2.70)	0.713
Anxious/distressed	44	1.78 (1.26–2.30)	47	2.54 (1.83–3.26)	0.107
Post-operative complication - wound	55	2.23 (1.65–2.81)	34	1.84 (1.23–2.45)	0.435
Collapsed	49	1.99 (1.44–2.54)	38	2.06 (1.41–2.70)	0.957
Skin cyst	60	2.43 (1.82–3.04)	25	1.35 (0.83–1.88)	**0.016**
Pyoderma	46	1.86 (1.33–2.40)	35	1.89 (1.27–2.52)	> 0.999
Post-operative complication – non-wound related	37	1.50 (1.02–1.98)	31	1.68 (1.09–2.26)	0.732
Alopecia	48	1.94 (1.40–2.49)	18	0.97 (0.53–1.42)	**0.014**
Allergic skin disorder	41	1.66 (1.16–2.17)	23	1.24 (0.74–1.75)	0.321
Entropion	54	2.19 (1.61–2.77)	9	0.49 (0.17–0.80)	**< 0.001**
Pruritus	32	1.30 (0.85–1.74)	31	1.68 (1.09–2.26)	0.366
Cruciate disease	37	1.50 (1.02–1.98)	25	1.35 (0.83–1.88)	0.787
Thin	41	1.66 (1.16–2.17)	19	1.03 (0.57–1.49)	0.104
Urinary incontinence	38	1.54 (1.05–2.03)	22	1.19 (0.70–1.68)	0.402
Dermatitis	35	1.42 (0.95–1.88)	22	1.19 (0.70–1.68)	0.608

*CI confidence interval. ** Two-proportion z-test comparing prevalence between Molossoid and non-Molossoid with P-values < 0.05 shown in bold. *n* = 4,316

Among the 21 most common grouped-level disorders of giant breed dogs overall, Molossoid had a statistically significantly higher prevalence of 4/21 (19.05%) (heart, female reproductive, mass, aural) and statistically significantly lower prevalence of 3/21 (14.29%) (overweight/obesity, behavioural, dental). No statistically significant difference in prevalence was detected in the remaining 14/21 (66.67%) disorders (Table 6).

**Table 6:** Prevalence of the most common disorders at *grouped-level diagnostic precision* in giant dog breeds overall broken down by Molossoid (*n* = 2,468) and non-Molossoid (*n* = 1,848) dog breeds under primary veterinary care at practices participating in the VetCompass™ Programme in the UK from January 1st to December 31st, 2019. *CI confidence interval. ** Two-proportion z-test comparing prevalence between Molossoid and non-Molossoid with P-values < 0.05 shown in bold. *n* = 4,316

Grouped-level disorder	Molossoid no. affected	Molossoid prevalence % (95% CI*)	Non-Molossoid no. affected	Non-Molossoid prevalence % (95% CI*)	*P*-value**
Dermatological	408	16.5 (15.01-18.0)	278	15.0 (13.4–16.7)	0.200
Musculoskeletal	349	14.1 (12.8–15.5)	234	12.7 (11.2–14.2)	0.173
Aural	397	16.1 (14.6–17.5)	119	6.44 (5.32–7.56)	**< 0.001**
Enteropathy	293	11.9 (10.6–13.2)	218	11.8 (10.3–13.3)	0.977
Behavioural	217	8.79 (7.68–9.91)	246	13.3 (11.8–14.9)	**< 0.001**
Dental	171	6.93 (5.93–7.93)	238	12.9 (11.4–14.4)	**< 0.001**
Overweight/obesity	167	6.77 (5.78–7.76)	179	9.69 (8.34-11.0)	**0.001**
Ophthalmological	173	7.01 (6.00-8.02)	124	6.71 (5.57–7.85)	0.746
Mass	182	7.37 (6.34–8.41)	106	5.74 (4.68–6.80)	**0.038**
Claw/nail	149	6.04 (5.10–6.98)	138	7.47 (6.27–8.67)	0.071
Neoplasia	160	6.48 (5.51–7.45)	110	5.95 (4.87–7.03)	0.517
Traumatic injury	96	3.89 (3.13–4.65)	82	4.44 (3.50–5.38)	0.414
Complication associated with clinical care	86	3.48 (2.76–4.21)	59	3.19 (2.39–3.99)	0.659
Respiratory tract	81	3.28 (2.58–3.98)	61	3.30 (2.49–4.12)	> 0.999
Disorder undiagnosed	87	3.53 (2.80–4.25)	50	2.71 (1.97–3.45)	0.152
Heart	89	3.61 (2.87–4.34)	41	2.22 (1.55–2.89)	**0.011**
Anal sac	62	2.51 (1.89–3.13)	58	3.14 (2.34–3.93)	0.252
Female reproductive (females only)	63	2.55 (1.93–3.17)	29	1.57 (1.00-2.14)	**0.035**
Urinary	59	2.39 (1.79–2.99)	32	1.73 (1.14–2.33)	0.166
Collapsed	49	1.99 (1.44–2.54)	38	2.06 (1.41–2.70)	0.957
Thin	51	2.07 (1.51–2.63)	29	1.57 (1.00-2.14)	0.278

### Mortality

In total, 1,068 (24.75%) of the giant breed dogs were recorded as having died during the available clinical records. The median age at death was 8.94 years (IQR 6.96–10.9, range 0.00–16.1). Females (*n* = 507, median longevity 9.31 years, IQR 7.35–11.4, range 0.00–15.4) lived statistically significantly longer than males (*n* = 556, median 8.49 years, IQR 6.53–10.6, range 0.00–16.1) (Mann-Whitney U test, *P* < 0.001). Median age at death within each breed ranged from 4.77 years in the Tibetan Mastiff to 12.7 years in the Black Russian Terrier (Table 7). Of 993 (93.0%) deaths with a recorded method of death, 872 (87.8%) deaths involved euthanasia and 121 (12.2%) deaths were unassisted. Molossoid breed dogs (*n* = 674, median longevity 8.42 years, IQR 6.82–10.2, range 0.00-15.9) lived statistically significantly shorter than non-Molossoid dogs (*n* = 394, median 9.97 years, IQR 7.43–12.1, range 0.00-16.1) (Mann-Whitney U test, *P* < 0.001).

**Table 7:** Age at death in giant dog breeds under primary veterinary care at practices participating in the VetCompass™ Programme in the UK from January 1st to December 31st, 2019. *N* = 1,068

Breed	No.	Median age (years) at death
Akita	74	11.3
Alaskan Malamute	136	11.2
American Akita	36	10.8
Anatolian Karabash Shepherd Dog	4	10.5
Bernese Mountain Dog	63	7.7
Black Russian Terrier	3	12.7
Bloodhound	2	6.32
Borzoi	9	9.26
Bull Mastiff	85	9.09
Dogue de Bordeaux	229	7.71
Estrela Mountain Dog	3	11.8
Giant Schnauzer	22	10.7
Great Dane	113	8.90
Great Swiss Mountain Dog	3	11.2
Irish Wolfhound	31	7.24
Leonberger	28	9.25
Mastiff	56	9.64
Neapolitan Mastiff	7	6.77
Newfoundland	70	10.3
Otterhound	2	9.03
Pyrenean Mountain Dog	11	11.0
Saint Bernard	64	6.76
Scottish Deerhound	13	9.23
Tibetan Mastiff	4	4.77

A biomedical cause of death was reported for 597 (55.9%) deaths. The most common causes of death at grouped-level precision were neoplasia (*n* = 132, 12.4%, 95% CI: 10.4–14.3), collapse (*n* = 101, 9.46%, 95% CI: 7.70–11.2) and heart disorder (*n* = 44, 4.12%, 95% CI: 2.93–5.31) (Table 8).

**Table 8:** Mortality reported at *grouped-level diagnostic precision* for disorders with 3 or more recorded deaths in giant dog breeds (*n* = 1,068)) under primary veterinary care at practices participating in the VetCompass™ Programme in the UK from January 1st to December 31st, 2019

Grouped-level disorder	Count	Percent (95% CI*)	Median age (years) at death
Disorder not diagnosed	471	44.10 (41.1–47.1)	9.13
Neoplasia	132	12.36 (10.4–14.3)	8.47
Collapsed	101	9.46 (7.70 − 11.2)	10.8
Heart disorder	44	4.12 (2.93–5.31)	8.04
Behavioural disorder	38	3.56 (2.45–4.67)	4.76
Enteropathy	35	3.28 (2.21–4.34)	8.96
Musculoskeletal disorder	35	3.28 (2.21–4.34)	9.52
Poor quality of life	27	2.53 (1.59–3.47)	10.0
Brain disorder	25	2.34 (1.43–3.25)	6.30
Abdominal disorder	23	2.15 (1.28–3.02)	8.07
Respiratory tract disorder	17	1.59 (0.84–2.34)	8.69
Mass	16	1.50 (0.77–2.23)	9.61
Spinal cord disorder	13	1.22 (0.56–1.87)	9.01
Haematopoietic disorder	12	1.12 (0.49–1.76)	7.55
Kidney disease	10	0.94 (0.36–1.51)	8.29
Female reproductive disorder	9	0.84 (0.44–1.59)	6.62
Appetite disorder	6	0.56 (0.26–1.22)	9.22
Skin disorder	6	0.56 (0.26–1.22)	8.00
Traumatic injury	6	0.56 (0.26–1.22)	7.12
Complication associated with clinical care	4	0.37 (0.15–0.96)	7.28
Incontinence	4	0.37 (0.15–0.96)	10.2
Lethargy	4	0.37 (0.15–0.96)	8.04
Neurological disorder	4	0.37 (0.15–0.96)	8.92
Pain	4	0.37 (0.15–0.96)	8.25
Shock	3	0.28 (0.10–0.82)	9.47
Thin	3	0.28 (0.10–0.82)	11.1
Other	16	1.50 (0.77–2.23)	5.00

*CI confidence interval

## Discussion

This is the first study to report on the demography, morbidity and mortality of giant breed dogs as an overall group in the UK. The findings show that giant breed dogs represent a relatively small but temporally consistent proportion of all dogs in the UK. The overall pattern of common disorders in giant breed dogs is not dissimilar to the patterns reported previously for dogs overall but the typical longevity of giant breed dogs in the current study is substantially shorter than previously reported for dogs overall [39, 47]. These new data and findings can contribute to wider discussions and debate on how far it is legally, ethically and welfare-wise acceptable to exaggerate the natural canine body shape in the quest by humanity for ever-expanding novelty in aesthetics of the companion dog [61].

Giant breed dogs are shown in the current study to represent 1.26% of all dogs in the UK, with consistent annual proportional birth rates from 2013 to 2019 suggesting stable demand for ownership. Previous work has shown that our human preferences for dog ownership are complex and are driven by different and unique belief patterns within each individual human and/or family seeking to own a dog [62–64]. The factors that contribute to these belief patterns are manifold and include owners’ self-imagery, *Kindchenschema* (preference for an infantile appearance), typical canine body shape, human-like attributes (e.g. coloured irises, upturned mouth commissure suggestive of smiling), calm and obedient, non-aggressive and safe, being healthy, being unhealthy, affectionate, medium-sized, non-shedding coat, being a puppy, being a rescue, prior experience with that breed, social media and advertising, and a long list of other human desires that even include availability of the dog for impulse purchase on a whim [65–72]. Across dogs overall over the past two decades, the combined effects from these ownership drivers is reported to have had substantial effects on overall companion dog demography with strong shifts towards owning smaller sized dogs, brachycephalic dogs (e.g. French Bulldog) and designer-crossbreed dogs (e.g. Cockapoo) [2, 73, 74]. However, the consistent annual proportional birth rates for giant dogs suggest this giant-size demographic of dogs holds low volatility in terms of overall ownership preferences. However, it is worth noting that that the relatively shorter lives of giant breed dogs compared to non-giant breed dogs could have introduced some survival bias for the annual proportional birth rates reported here. A decreasing proportion of dogs born in earlier years may have remained alive to be included in the current single year dataset of dogs in 2019 and so it is possible that this selection bias from loss to inclusion for giant breed dogs born in earlier years may have masked a true declining proportional ownership of giant breed dogs overall [75].

It is notable that although generic grouping terms for dogs such as giant, brachycephalic, chondrodystrophic and extreme conformation are commonly and comfortably used by breeding, legislative, academic, veterinary, charity and wider public communities, these terms are rarely matched to formal universally-agreed lists of breeds [3, 76–80]. This lack of clarity can lead to wide confusion both for the public who may wish to use these grouping terms to assist with responsible acquisition decisions (e.g. to understand which breeds or types of dogs are generally considered to have extreme morphotypes so they can avoid acquiring a dog with an extreme conformation in line with international welfare guidance [77]) and also for researchers aiming for comparability in their research across studies worldwide [81]. The current authors have previously encountered this challenge when reporting on disorder risk in brachycephalic dogs overall where the absence of a formal list of brachycephalic dog breeds required *de novo* generation of such a list within the VetCompass research programme that has since been accepted as a standard in other research [14, 74, 82, 83]. A similar lack of a standardised list of giant dog breeds required *de novo* generation of a list of giant breeds in conjunction with the Royal Kennel Club for the current study. This list of giant breeds reflected the knowledge and beliefs of the current researchers at the time of this UK study but future researchers at later times or in non-UK locations may opt to apply different lists. To reduce such confusion in the future, it would be helpful for standard lists of major demographic categorisations of dog breeds to be internationally agreed.

Although no specific bodyweight range is formally defined for giant breeds, dog breeds with a typical adult bodyweight greater than 45 kg are generally accepted as giant [12, 15, 16]. Interestingly, only 22 of the 29 breeds included as giant in the current study showed a median adult bodyweight above 45 kg. This apparent discordance may reflect frequently wide divergence between the written breed standards for pedigree show dogs that appear to promote moves towards increasingly extreme conformation and the wider population of dogs where more moderate and non-extreme (or less extreme) conformation may be preferred. For example, in the current study of giant breeds, the Hungarian Kuvasz showed the lowest mean adult bodyweight at just 31.4 kg but the RKC breed standard requires male dogs of this breed to weigh 40–52 kg, explaining why the Hungarian Kuvasz was included as a giant breed in the current study [3]. Similar discrepancy between breed standard bodyweights that are more extreme than the real-world bodyweights in the wider dog population have been reported previously for the Yorkshire Terrier (3.2 kg breed standard versus 5.06 kg real-world) and Chihuahua (1.8–2.7 kg breed standard versus 3.4 kg real-world). Although some of this difference breed standard and real-world bodyweights in these smaller size breeds could explained by high levels of overweight or obesity in the real-world dogs [84], the current and previous results do suggest that the levels of exaggeration desired in pedigree dog show standards are often out of line with wider public preferences and therefore warrant reconsideration of show standards towards more moderate ideals [67, 85, 86].

Overall, 73.8% of the giant breed dogs under primary veterinary care in the current study had at least one disorder recorded during 2019. This proportion is higher than the 65.8% of dogs of all types recorded with at least one disorder recorded during 2016 in a previous VetCompass study that used the same methods and data source as the current work [47]. This could suggest that giant breed dogs have poorer health status on average than dogs overall and therefore a higher proportional need for veterinary care to focus on clinical disorder management rather than for routine prophylactic care. Alternatively, it is possible that accessibility of veterinary care may be more logistically and financially challenging for owners of giant dogs and that the current findings could be explained by lower levels of prophylactic care being sought because of these challenges rather than truly higher proportional clinical morbidity in giant dogs [87–90]. A prospective study of owned dogs in the wider population would be required to explore differential barriers to accessing veterinary care between giant and other dog types to fully interpret the high proportion of disorder-based care identified in the current study.

The most common precise-level disorders identified in giant breed dogs in the current study were otitis externa (8.16%), overweight/obesity (8.02%), aggression (5.56%), dental disease (5.51%) and overgrown nail(s) (5.38%). This list of the most common disorders in giant breed dogs is similar to those previously recorded in dogs of all types during 2016 in a previous VetCompass study where the most prevalent disorders were periodontal disease (12.52%), otitis externa (7.30%), obesity (7.07%), overgrown nail(s) (5.52%) and anal sac impaction (4.80%) [47]. These most common disorders in both giant and all dog types generally reflect basic biological ageing, degenerative and immunological processes common to all canines that are largely independent of breed or body size and therefore it is unsurprising that four of the five most common disorders were shared between both giant and all dog types [91–93]. However, it is notable that the prevalence of these shared common disorders varied between the giant versus all dog types. Lower prevalence of dental disease in the giant breed dogs compared to all dogs was supportive of previous reports of protection to dental disease in larger sized dogs [94–96]. In the opposite direction, giant breed dogs showed higher prevalence compared to all dogs for otitis externa [97] and overweight/obesity [84] that was in line with similar previous reports. These contrasts suggest differential health risks between giant and other dog types that are disorder specific and do not support an overall health disadvantage in either group of dogs.

Aggression was one of the two notable differences among the lists of the top five disorders, being the third most common disorder recorded in the current study of giant breed dogs in 2019 with a prevalence of 5.56%. This prevalence was more than double the prevalence of 2.24% recorded in dogs overall in 2016 [47]. Although it is possible that the behavioral threshold for veterinarians to record dogs as aggressive had changed over this 3-year period, this finding of higher recorded proportional aggression in giant breed dogs still aligns poorly with much of the previous research that reported higher proportional aggression in smaller sized dogs [35, 36, 98, 99]. It is possible that this discordance reflects implicit bias that varies in effect between study designs, whereby their large body size and perception of threat mean that equivalent behavioural suites are considered as more aggressive for giant dogs compared with smaller dogs [100]. Media portrayal of giant breeds such as Mastiff or Akita as dangerously aggressive may also have shaped such public and veterinary perceptions and bias [101, 102]. Previous work has shown that growls with the same acoustic cues were rated as more aggressive from larger dogs than from smaller dogs [103]. Given the national UK debate over recent decades on breed-specific legislation firstly in relation to the Dangerous Dogs Act 1991 [104–107] and more recently in relation to XL Bully dogs [108, 109], the current results support an ongoing real or perceived issue with heightened aggression in giant breed dogs [110].

Molossoid refers to a subset of giant breed dogs with a related ancestry that were used historically to guard and protect properties and livestock [111]. Molossoid breeds are included as Group 2 breeds by Fédération Cynologique Internationale (FCI) and include Anatolian Karabash Shepherd Dog, Bull Mastiff, Dogue de Bordeaux. Estrela Mountain Dog, Great Dane, Leonberger, Saint Bernard, Mastiff, Neapolitan Mastiff, Newfoundland, Pyrenean Mastiff, Pyrenean Mountain Dog, Tibetan Mastiff and Turkish Kangal [112]. Molossoid dogs are typically characterised with a comparatively large head, shortened muzzle, heavy bone and often thick and wrinkled skin [111]. Dogs from Molossoid breeds were previously reported to have higher overall morbidity and shorter life expectancy compared to non-Molossoid dogs, with Mastiff breeds specifically reported with a median longevity of 8 years [113–115]. The current results did not provide any evidence of differential overall health status between Molossoid and non-Molossoid dogs within the giant breeds, with an almost even split of the most common disorders being more, less or equally prevalent in Molossoid and non-Molossoid dogs. However, median longevity was shorter in Molossoid breed dogs (8.42 years) than non-Molossoid dogs (9.97 years). Given that the median adult bodyweight for Molossoid giant breeds (55.0 kg) was substantially heavier than for non-Molossoid giant breeds (41.3 kg), this shorter lifespan in Molossoid giant breeds may largely reflect the well-documented shortening of life expectancy in dogs as bodyweight increases [38–41, 44, 116–119].

The current study reported a median life expectancy of 8.94 years for giant breed dogs overall that is substantially shorter than the median longevity of 12.0 years previously reported within a VetCompass of dogs overall in England [39]. This 25% reduction in average life expectancy in giant breed dogs aligns with research reported in many countries over several decades showing that adult size of dog is inversely correlated with lifespan [38–41, 44, 116–119]. Longevity within individual giant breeds has also previously been reported as curtailed relative to smaller dogs: Leonberger at 7.7 years [120], Irish Wolfhound at 6 to 8 years [121, 122], Mastiff at 8.0 years [123] and Bernese Mountain Dog at 8.4 years [124]. Given that the dog breed concept is a relatively recent human invention to fit our human desires for novel dog types with working, tradition, social, aesthetic or other appeal, any substantial health, welfare or lifespan cost to the dog that is driven by breed criteria raise challenging ethical and moral conundrums [125]. On the one hand, if a shorter life in giant dog breeds just reflects an accelerated aging process but with an equivalent proportional healthspan (i.e. proportion of life that is considered ‘a good life’) to smaller sized dogs, it could be argued that the overall welfare cost at a population level for giant breed dogs is nullified [126–128]. However, if their more rapid ageing brings both a proportionally shorter healthspan as well as an absolutely shorter lifespan, then the health and welfare cost to the dog from our human desires to own giant dogs could be considered deontologically ethically unacceptable [129].

Physical size is one of the several key phenotypic criteria used to distinguish between dog breeds, with height to the withers (cm or inches) or adult bodyweight (kg or lbs) included in many breed standards worldwide [76, 130, 131]. However, there is increasing challenge to the ethics and welfare of selection for ever-bigger versions of dogs that is counter to innate health principles of only selecting for phenotypes with a full capacity to live a life and lifespan typical for the species overall [10, 13, 61, 78, 129]. Extreme conformation in dogs is defined as a physical appearance that has been so significantly altered by humankind away from the ancestral natural canine appearance that affected dogs commonly suffer from poor health and welfare, with negative impacts on their quality and quantity of life [77]. The results from the current study showing higher proportions of giant dogs recorded with at least one disorder annually and shorter lifespans in the giant breeds provide some evidence to support wider conversations of considering what levels of giantism in dogs should meet the threshold as an extreme conformation. Over time, setting a measurable threshold for extreme giantism can help welfare-focused breeders to better understand their legal and ethical responsibilities. Schedule 6 [5, 6] of The Animals Welfare (Licensing of Activities Involving Animals) (England) Regulations (LAIA Regs) 2018 states “*No dog may be kept for breeding if it can reasonably be expected*,* on the basis of its genotype*,* phenotype or state of health*,* that breeding from it could have a detrimental effect on its health or welfare or the health or welfare of its offspring”*, with similar legislation existing in Scotland and Wales [132–134]. Similar legislation to restrict breeding from dogs and cats with extreme conformation is being enacted in the European Union [135].

It is worth noting that the current paper conducted multiple individual statistical tests but P-values were not adjusted for this multiple testing. Consequently, the possibility of an inflated type I error rate (false positive) should be considered when interpreting statistically significant findings from any individual test [60]. The results of individual tests are therefore best viewed as hypothesis-generating and requiring confirmation in later independent datasets or future prospective studies where the specific question of interest [136].

The current study had some limitations. Disorders were recorded in the study as a binary variable (present or not present in the year of the study) for each dog and did not account for severity or duration that would have contributed to a deeper understanding of the fuller welfare burden [137]. Recorded disorder diagnoses relied on the clinical acumen and note-taking habits of the veterinary teams and therefore may have under-estimated true disorder status [138]. Some disorders were recorded as formal biomedical diagnosis terms (e.g. otitis externa) but others were recorded used broader clinical descriptors (e.g. diarrhoea). However, this reflects the contextualised care nature of primary veterinary care where resolving the clinical issue is often a greater priority than reaching an unnecessary final specific diagnosis [139–141].

## Conclusions

Giant breed dogs represent a relatively small (1.26%) but consistent proportion of all dogs owned in the UK. The current results provide some evidence of higher disorder burden and shorter lifespans in giant breed dogs overall compared to the wider population of dogs that raises concerns about negative impacts on both the quality and quantity of life resulting from our human selection for giantism in dogs. These findings suggest value from considering setting international welfare-based limits for height and bodyweight exaggerations selected in giant dogs.

## Data Availability

The dataset supporting the conclusions of this article are openly available on Figshare 10.6084/m9.figshare.30964531.

## Abbreviations

CI: Confidence interval
EHR: Electronic health record
IQR: Interquartile range
NIH: National Institutes of Health
RKC: Royal Kennel Club
